# Just how plain are plain tobacco packs: re-analysis of a systematic review using multilevel meta-analysis suggests lessons about the comparative benefits of synthesis methods

**DOI:** 10.1186/s13643-018-0821-7

**Published:** 2018-10-05

**Authors:** G J Melendez-Torres, James Thomas, Theo Lorenc, Alison O’Mara-Eves, Mark Petticrew

**Affiliations:** 1 DECIPHer, School of Social Sciences, Cardiff University, Cardiff, UK; 2 EPPI-Centre, Social Science Research Unit, UCL Institute of Education, London, UK; 3 Centre for Reviews and Dissemination, University of York, Heslington, UK; 4 Department of Social and Environmental Health Research, London School of Hygiene and Tropical Medicine, London, UK

**Keywords:** Systematic review, Meta-analysis, Narrative synthesis

## Abstract

**Background:**

Comparisons between narrative synthesis and meta-analysis as synthesis methods in systematic reviews are uncommon within the same systematic review. We re-analysed a systematic review on the effects of plain packaging of tobacco on attractiveness. We sought to compare different synthesis approaches within the same systematic review and shed light on the comparative benefits of each approach.

**Methods:**

In our re-analysis, we included results relating to attractiveness in included reports. We extracted findings from studies and converted all estimates of differences in attractiveness to Cohen’s *d*. We used multilevel meta-analysis to account for clustering of effect sizes within studies.

**Results:**

Of the 19 studies reporting results on attractiveness, seven studies that included between-subjects analyses could be included in the meta-analysis. Plain packs were less attractive than branded packs (*d* = − 0.59, 95% CI [− 0.71, − 0.47]), with negligible but uncertain between-studies heterogeneity (*I*^2^ = 0%, 95% CI [0.00, 70.81]) and high within-study heterogeneity (*I*^2^ = 92.6%, 95% CI [91.04, 93.90]).

**Conclusions:**

The meta-analysis found, similar to the narrative synthesis, that respondents typically rated plain packaging as less attractive than alternative (e.g. branded) tobacco packs. However, there were several trade-offs between analysis methods in the types and bodies of evidence each one contained and in the difference between partial precision and breadth of conclusions. Analysis methods were different in respect of the role of judgement and contextual variation and in terms of estimation and unexpected effect modification. In addition, we noted that analysis methods were different in how they accounted for heterogeneity and consistency.

**Electronic supplementary material:**

The online version of this article (10.1186/s13643-018-0821-7) contains supplementary material, which is available to authorized users.

## Background

The debate on the relative merits of narrative synthesis and meta-analysis is a well-rehearsed one in the field of systematic review methods [1]. Yet to our knowledge, a ‘within-review’ examination of the comparative benefits of each synthesis method has not been undertaken recently. Here, we re-analyse findings from a systematic review and narrative synthesis on plain packaging of tobacco. Our objectives in re-analysing these findings were to (a) compare different synthesis approaches within the same policy-relevant systematic review and (b) shed light on the comparative benefits of each approach, including with respect to policy implications of choice of synthesis methods.

### The methodological debate

Approaches to the synthesis of quantitative evidence take a variety of forms, broadly categorised as narrative approaches, which ‘tell the story’ of the evidence in a systematic review [2], and meta-analytic approaches, which pool studies statistically to yield a combined weighted effect. Though the latter can provide evidence relating to average effect and associated imprecision of that effect, it may obscure contextual patterns in the data [3]. In contrast, narrative synthesis, which involves a descriptive analysis to highlight similarities in findings in included studies and characteristics, can highlight these contextual patterns but relies to a greater extent on researcher judgement [4]. Narrative synthesis has also been deemed to be susceptible to ‘vote counting’, which, in its most unreliable form, involves tallying the number of statistically significant results in each direction to decide whether on balance an intervention is effective or not—which is more often than not misleading [5]. Meta-analysis may also be underused in systematic reviews due to concerns about heterogeneity and the suitability of the evidence for meta-analysis, even if a meta-analysis would be of utility in understanding heterogeneity and an ‘average’ effect [6].

Previous work by our group comparing narrative syntheses and meta-analyses has suggested that these two approaches may be answering different questions via different approaches, rather than answering the same questions using the typically preferred method (meta-analysis) or its ‘backup’ (narrative synthesis) [1]. In this work, we described that narrative syntheses and meta-analyses use different modes of reasoning to answer related but distinct questions; in the case of narrative synthesis, the question most often asked is ‘what is going on here?’ or ‘what picture emerges?’ whereas in meta-analysis, the question most often asked is ‘does it work and how well?’ and ‘will it work again?’. We described these two types of questions as relating primarily to a practical and configurational mode of reasoning embedded in the sense-making aspects of narrative synthesis, and to a predictive and inferential mode of reasoning embedded in the pooling and testing aspects of meta-analysis. Distinguishing between these two modes of reasoning is important because, for example, reviewers who set out to do meta-analysis but ultimately undertake narrative synthesis may be answering different questions than those they originally sought to answer. However, to our knowledge, no recent review has been ‘re-analysed’ using a different, non-statistical synthesis method than was originally employed to examine if these two different methods yield different results. This is important as it might provide insights into how the choice of synthesis method for what would ostensibly be a similar question can influence presented findings. To address this gap, we used data from a previously published systematic review of 37 studies of plain standardised packaging of tobacco products [7, 8].

### Policy context

Plain packaging of tobacco products, which was introduced as part of the World Health Organization Framework Convention on Tobacco Control, is intended to reduce the demand for tobacco products by removing an opportunity for marketing [9]. The focal systematic review was originally commissioned by the UK Department of Health in 2013 to inform policy development on standardised packaging of tobacco commissioned. At a later point in the policy process, an independent examination of the issue was commissioned from Sir Cyril Chantler, who obtained examination of the full report of the focal systematic review [7] by two independent academic groups, both of which agreed as to the high standard to which the systematic review was undertaken. However, the systematic review met with unsurprising criticism, including methodological criticism, from the industry. For example, Japan Tobacco International commented that the focal systematic review ‘is simply a narrative study. It neither achieves the objectives set by the [UK Department of Health], nor is it a “meta-analysis”. It is not clear what value the Systematic Review can add at all, over and above each of the individual underlying studies it reviews’ [10]. The company goes on to note that ‘the authors have failed in their attempt to produce any quantifiable evidence as to any impact of plain packaging’.

### The focal systematic review

This systematic review [7], which aimed to summarise all primary research relating to plain tobacco packaging, narratively synthesised evidence from 25 primary quantitative research studies (18 cross-sectional experimental studies including within-subjects or between-subjects comparisons, three cross-sectional non-experimental studies, three mixed-methods studies and one intervention study) relating to comparisons between standardised packaging of tobacco products and branded tobacco products and addressing benefits of plain packaging anticipated in the Framework Convention on Tobacco Control. The narrative synthesis was undertaken across seven domains: attractiveness of standardised packs, perceived quality and taste of cigarettes sold in standardised packs, smoker identity associated with standardised packs, salience of health warnings, perceptions of tar or nicotine levels, perceptions of harmfulness and ease of quitting. It was published in full as a report including all 37 studies [7] and subsequently condensed into a journal article focusing on these 25 studies [8]. We cite each as appropriate. In the original report, the authors describe the choice of synthesis methods as such:

The possibility of combining the studies statistically in a meta-analysis was explored. Given the diversity of research questions addressed in the included studies, most of which vary on at least four dimensions (typically populations, interventions, comparators, and outcomes), it is not appropriate to conduct a quantitative synthesis of these studies. In other words, there was too much heterogeneity [7].

Anticipating criticisms of this approach, the authors described that they sought to avoid ‘vote counting’ in the narrative review [7, 8]. They examined both significance and direction of effect, as well as presenting direction of effect in tables in the text, and presented effect sizes for certain outcomes. They also examined studies in subgroups relating to smoking status and age.

In the focal systematic review, 19 of the subset of 25 included studies examined attractiveness of plain packages as compared to branded packages. These studies included a total of 27,166 participants and represented a range of designs, including between-subjects designs (e.g. cross-sectional designs where participants were randomised to view different packs), within-subjects designs (e.g. where participants viewed several different packs and selected the most attractive) and ‘prevalence’ designs (e.g. where the percentage of respondents selecting a plain package as less attractive was compared against a presumed probability distribution). The narrative synthesis in the original review noted that ‘findings were highly consistent, with all studies reporting that standardised packs were considered less ‘appealing’, ‘attractive’, ‘cool’, ‘stylish’ and ‘attention-grabbing’ than branded equivalent packs’ [8]. Comparison of studies by subgroups and of subgroup analyses presented within papers led the authors to suggest that ‘non-smokers and younger respondents were more affected by standardised packaging’, that is, that these groups found plain packages even less attractive than smokers or older respondents, respectively [8].

To understand the implications of different synthesis methods within the same study, we included studies from the original review reporting results for attractiveness of standardised packs and, where possible, reanalysed findings using multilevel meta-analysis. We compared the findings of the narrative synthesis and the meta-analysis, and we used this comparison to consider the value of the different approaches, including trade-offs between different synthesis methods.

## Methods

In our re-analysis, we included results relating to attractiveness in included reports. Where effect sizes were presented in a usable metric, we transformed them into Cohen’s *d*, a measure of standardised mean difference that is used when conceptually related outcomes are reported on different scales [11]. Where necessary, we used the logistic transformation to convert odds ratios derived from binary outcomes into Cohen’s *d* or used test statistics such as *F* tests to derive a standardised mean difference. In several studies, results were presented in aggregate across several comparisons to create a continuous scale and in different subgroups. Thus, in the original report, the approach taken was to combine ‘across subgroups that were not of interest such as gender groups, to get the frequency across all people for the actual group of interest’ [7]. In this analysis, we used disaggregated estimates where these were available to better understand statistical heterogeneity, and in contrast to the original report, we used standardised mean differences to render effect sizes on the same scale. These disaggregated estimates frequently related to plain vs. branded packaging within specific brands, but also to different branded pack characteristics (e.g. the presence of cigarette type descriptors). In the original report [7], studies were appraised in triplicate using a set of seven items relating to sampling, data collection and data analysis, with a positive score on all seven items indicating a high-quality study, on four to six items indicating a medium-quality study and zero to three items indicating a low-quality study.

Because included studies reported multiple relevant effect sizes, we used a multilevel meta-analysis method to combine effects, with random effects at both the study level and at the effect size level [12]. In this model, effect sizes are nested within studies. ‘Clustering’ effect sizes in this way accounts for non-independence of errors and has been shown to work well in practice [12]. One benefit of this method is that it allows for the partitioning of heterogeneity into within-study and between-studies *I*^2^, indicating if variation between effect sizes is primarily within studies, between studies or due to sampling error. Variance components at the study level and at the effect size level were estimated using restricted maximum likelihood. We did not assess publication bias due to the low number of studies included in the analysis.

All analyses were carried out using the R package metafor, using function rma.mv (Viechtbauer, 2014). Graphs were produced using Stata v.14 (Statcorp, College Station, TX).

## Results

**Fig. 1:**
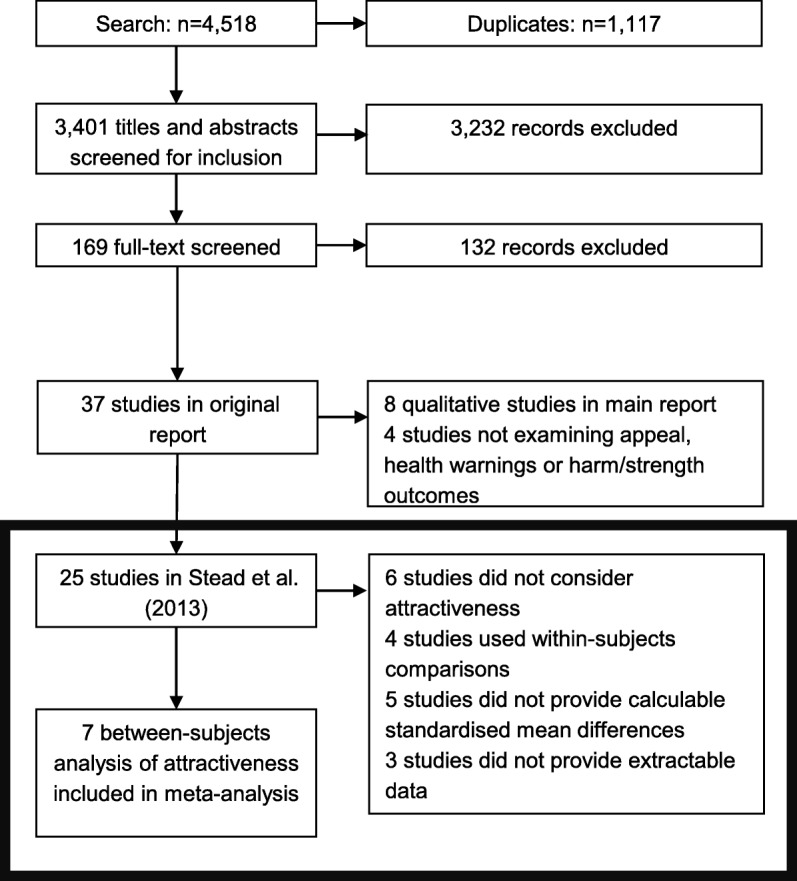
PRISMA flowchart. The current paper focuses on the stages described in the box

Of the 19 studies reporting results on attractiveness, only seven studies [13–19] covering 5365 participants and including between-subjects analyses (i.e. comparing ratings from those exposed to plain packs against ratings from those exposed to branded packs) were included in the meta-analysis (see Fig. 1). This was for several reasons. Four studies used within-subjects comparisons, which could not be readily rendered on the same scale as studies using between-subjects comparisons, and adequate information was not provided in these studies to facilitate this transformation. For example, one study used a ‘difference measure’ within subjects to compare likelihood of choice of packs [20]. An additional five studies did not present effect sizes for the relationship between plain packaging and attractiveness in a metric that could be converted to standardised mean differences between plain and branded tobacco packs. For example, one study compared the prevalence of respondents choosing one plain pack as less attractive against a null distribution of 50% prevalence [21]. Finally, three studies did not present extractable data. For example, one study presented an *F* test and significance threshold (*p* < 0.05) without degrees of freedom [22] (see Additional file 1 for full details of excluded studies).

**Fig. 2:**
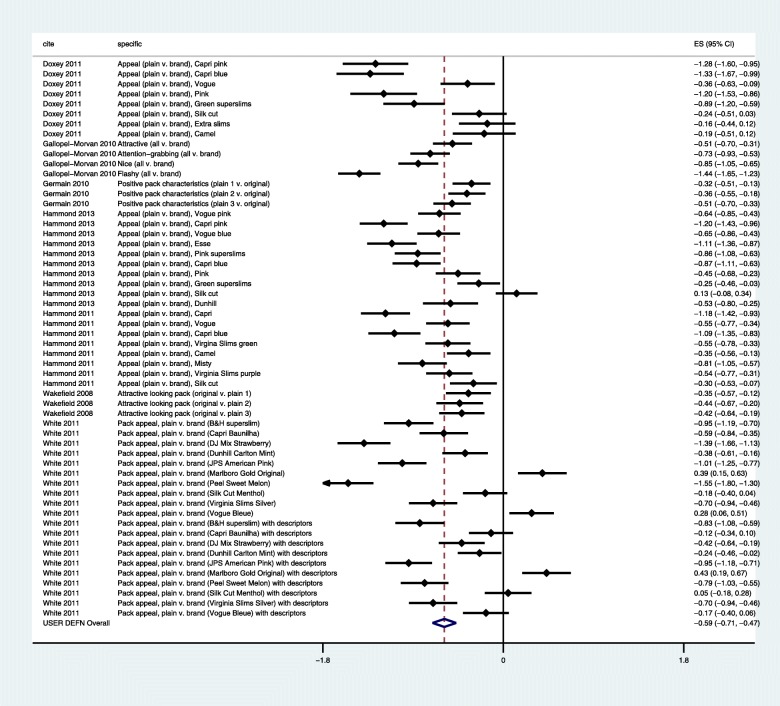
Multilevel meta-analysis of attractiveness in plain tobacco packages. Units are in Cohen’s *d*. Negative estimates indicate decreased attractiveness of plain packs

The seven included studies yielded 56 effect sizes (see Fig. 2). Four studies presented effect sizes for specific brands as part of the same experiments, two studies presented effect sizes for specific types of plain packs and one study presented effect sizes for specific aspects of attractiveness. Multilevel meta-analysis with random effects both within and between studies suggested that plain packs were less attractive than branded packs (*d* = − 0.59, 95% CI [− 0.71, − 0.47]). Converted to an odds ratio of 2.91, this suggests that plain packs had odds nearly three times higher of being deemed unattractive as compared to branded packs. Examination of variance components in the multilevel meta-analysis model showed that between-studies heterogeneity was negligible (*I*^2^ = 0%, 95% CI [0.00, 70.81]), albeit with wide confidence intervals where we could not exclude high heterogeneity, whereas within-study heterogeneity was high (*I*^2^ = 92.6%, 95% CI [91.04, 93.90]). In the original report [7], each of the seven studies was appraised as being of medium quality.

## Discussion

Using statistical meta-analysis methods, we re-analysed findings from a systematic review that had originally been presented using narrative synthesis and found, similar to the narrative review, that respondents typically rated plain packaging as less attractive than alternative (e.g. branded) tobacco packs. However, each method has a distinct value as captured by the trade-offs between each method. First, we will discuss these trade-offs, before reviewing strengths and limitations of our particular investigation and then offering suggestions for the practice of systematic reviewing and for future research.

### Broad conclusions vs. partial precision

The first and most immediate trade-off between the two syntheses was in the types and bodies of evidence each one contained. The narrative synthesis was able to include a variety of study types, each with an appropriate design for answering the question at hand. This facilitated the inclusion of studies published before reporting guidelines that would support extraction and inclusion of study effects, but it also facilitated the inclusion of modern studies conducted using within-subjects comparisons, which could not readily be rendered in a scale commensurate with between-subjects comparisons [23]. We ultimately had to exclude these studies, as well as studies using ‘non-standard’ effect metrics, from the meta-analysis. This is an important distinction from criticisms traditionally levelled at narrative synthesis, namely, that narrative syntheses may treat studies of uneven quality or differential ability to estimate causal relationships as being equals with ‘true’ randomised studies. The studies we excluded were not necessarily of differential quality, but they did differ in design characteristics from the studies we were able to include in our meta-analysis.

While both narrative syntheses and meta-analyses can offer conclusions with definitiveness—that is, with a definitive view as to whether the intervention is effective or not for a given outcome—only meta-analyses can offer *precision*, which we take here to be the uncertainty associated with a particular intervention effect. While the meta-analysis only included seven studies, it was able to estimate with an associated precision the expected difference in attractiveness associated with plain packaging from a partial set of relevant studies. However, this precision may be incomplete in relation to the wider body of evidence in that it only accounts partially for the evidence included. Because we were unable to generate meaningful effect sizes from these studies that were commensurate with the effect sizes generated from the meta-analysable studies, we could not evaluate the counterfactual scenario of a meta-analysis including all 19 studies. The 12 studies may have enhanced the precision of the pooled estimate, assuming similarity in magnitude of effect to the studies we included, or if effects from these studies were to skew in a different direction than the pooled studies, the pooled estimate may be misleadingly precise.

Moreover, while the conclusions of the narrative synthesis are based on a variety of evidence types that can make different knowledge claims (causal and associative), the meta-analysis synthesised evidence designed to make causal claims. This does not necessarily mean that the meta-analysis is lacking; however, if we are only interested in the causal relation between the manipulated variable (i.e. the appearance of the package) and the outcome (i.e. perceived attractiveness of the package), then the conclusions of the meta-analysis should not be seen as incomplete in addressing that question (although in this case, the internal validity of the results based on the study design, such as randomisation to conditions, is very important in establishing the strength of the causal claim). In other words, the ‘completeness’ of the narrative synthesis does not necessarily mean the ‘incompleteness’ of the meta-analysis—they are simply ‘complete’ answers to questions with different foci.

### Consistency of findings vs. statistical heterogeneity

Another key trade-off between the two synthesis methods related to how each method addresses the ‘spread’ of results in included studies. While the narrative synthesis inspected findings for consistency—that is, for the similarity of direction of effect—the meta-analysis was able to quantify heterogeneity by using *I*^2^. In this case, consistency and heterogeneity are related, but not identical. In the narrative synthesis, consistency indicated whether or not studies produced results on the same side of the line, that is, whether studies were consistently positive or negative. This is distinct from the ‘traditional’ view of vote counting, which at its most misleading fixates on null hypothesis significance testing rather than the direction and precision of effect. In contrast, the quantification of heterogeneity provides a basis for understanding the dispersion of results but cannot speak to consistency of effect direction in itself, and narrative synthesis is required to transform an ‘examination’ of dispersion into meaningful interpretation. In this case, the narrative synthesis found that included studies were consistent in their findings, while the calculation of *I*^2^ suggested there was little between-studies heterogeneity, albeit with wide confidence intervals. Indeed, an ancillary benefit of calculation of *I*^2^, and one often underutilised in meta-analyses, is the ability to consider the imprecision of heterogeneity estimates, that is, how ‘confident’ are we in a specific estimate of heterogeneity?

### Judgement and contextual variation vs. estimation and unexpected effect modification

A third trade-off relates to the opportunities each synthesis method affords for asserting similarities and differences across contexts. This trade-off flows from the previous two trade-offs identified. In the original narrative synthesis contained in the full report [7], narrative synthesis was chosen because of concerns over conceptual and methodological heterogeneity across different study types and questions. In contrast, meta-analysis was used in this study to better understand statistical heterogeneity by focusing on a narrower set of meta-analysable studies (and thus, a set of studies with less methodological heterogeneity than in the original review). Both types of synthesis arrived at the same conclusion: plain tobacco packaging is associated with a reduction in package attractiveness. However, each method generated ancillary observations. The narrative synthesis was able to assert a key generality across studies. This generality was that all studies arrived at similar conclusions regardless of sample or other contextual factors. This assertion could only have arisen from conceptual engagement with the context within which each study was conducted, as meta-analysis does not afford the opportunity to ‘formalise’ judgement about contextual difference and similarity. That is, narrative synthesis allows for more room for reviewer judgement about the relevance of contextual factors and the ability to explore consistency and generality across these contextual factors. Access to a greater body of studies than would necessarily be meta-analysable facilitates understanding consistency and generality, but also difference and variation, across contexts.

On the other hand, quantification of heterogeneity within and between studies in the multilevel meta-analysis suggested that there was lot of information *within* studies that could generate hypotheses for effect modification. Inspection of the effect sizes in the forest plot suggested that a key effect modifier could be brand or type of cigarette package. Results from the four studies where findings were disaggregated by brand suggested that aesthetic considerations could modify the relationship between plain packaging and attractiveness. This finding would not have been suggested but for the use of a forest plot and quantification of heterogeneity. In this way, graphical displays and meta-analysis can be helpful to ‘see the trees for the forest’. Furthermore, the meta-analysis allowed quantification of heterogeneity between studies. This was found to be low relative to the sampling error in the effect sizes (that is, *I*^2^ = 0%), albeit imprecise in its estimation. But while low heterogeneity is related to asserting consistency, it does not carry the same conceptual power as consideration of consistency in light of contextual variation.

### Implications in relation to the focal systematic review

As noted above, both analyses came to the same conclusions regarding the relationship between plain packaging of tobacco and attractiveness. However, the re-analysis presented here accounts for some of the potential issues attendant to narrative synthesis, but with significant limitations in its own right. One of the reasons the focal systematic review did not use meta-analysis was the diversity of relevant and equally informative bodies of evidence encompassing a variety of study designs. This will frequently be the case in reviews designed to inform public policy: they will not draw on a single best canonical study design, such as the randomised trial, to answer the question at hand.

### Implications for systematic reviewers

Moreover, as we have shown, restricting bodies of evidence to only studies that are meta-analysable requires trade-offs that may not be desirable in a systematic review that necessarily requires a liberal view of evidence to inform public policy. This is not an uncommon problem. For example, a recent systematic review of alcohol advertising restrictions [24] relied only on evaluations of marketing restrictions and concluded that evidence did not support the implementation of these restrictions. This disregarded the vast literature on the relationship between alcohol advertising and individual decisions, and failed to account for the role that alcohol advertising plays in shaping consumption decisions embedded within complex social systems [25]. In our case, meta-analysis was also not a panacea: despite our finding of relatively low between-study heterogeneity, we also found that this estimate was imprecise.

Moving forward, choice of synthesis method should be supported not only by the proposed uses of the evidence, but also by the nature of the evidence included. This seems a fairly obvious point, but our analysis has suggested additional considerations on evidence use and evidence types that previous guidance may have not brought to the fore. For example, where systematic reviews are to be used to inform modelling (e.g. in the health economics/decision analysis context), the choice between broad conclusions and partial precision might weight towards partial precision achieved via meta-analysis. In contrast, where equally valid types of evidence display heterogeneity of design and concept alongside possible statistical heterogeneity, the broad conclusions permitted by narrative synthesis may provide a more relevant answer to a review question. Similarly, where a review would most usefully assert generalities across types of evidence and contexts, a narrative synthesis is most appropriate. But where there is a need to assess and estimate the impact of moderators, meta-analysis may be a more suitable tool. It is likely that in some cases, systematic reviewers will wish to reap the benefits of both methods. For example, systematic reviews focused on health equity impacts of interventions frequently make use of two types of evidence: trials undertaken in disadvantaged groups as compared to trials undertaken in majority/non-disadvantaged groups, and subgroup analyses undertaken within trials [26]. Subgroup analyses undertaken within trials can be synthesised using harvest plots, which is a narrative synthesis-led tool, whereas comparison of trial effectiveness can be undertaken using meta-regression methods.

### Strengths and limitations

A strength of this analysis was the exhaustive and detailed search and appraisal undertaken in the primary systematic review [8], which was validated by independent academic groups. In addition, the research team’s careful use of narrative synthesis beyond simply ‘vote counting’ ensured that we were comparing a well-conducted narrative synthesis against a meta-analysis. However, this analysis was based on just one review, and further examples of narrative syntheses where meta-analysis could have been undertaken—and of meta-analyses, where narrative syntheses would also have been appropriate and defensible—could furnish additional information about the comparative value of the two methods. That is to say, this analysis, while suggestive of key considerations, is tentative and subject to revision as additional methodological learning and options arise in systematic review methodology. Future analyses could also consider the primary field of the review and the literature synthesised: whether from a more ‘traditional’ health technology assessment perspective or from a public health and complex intervention lens, whether the evidence is epidemiological or interventional; and whether the primary method used as the outset was meta-analysis or narrative synthesis. Finally, all reviews are subject to incomplete searches. Though authors did take an extensive approach to searching, it is possible that studies may have been missed.

### Further research

A small body of research has focused on the role of the judgement of systematic reviewers when faced with complex methodological and substantive decisions [27, 28] and on the role of reading for meaning in systematic reviews [29]. This investigation suggests that there is an opportunity to extend this work and better understand how reviewers choose synthesis methods at the point of combining studies. How do reviewers weigh up the different benefits and challenges associated with each method, and how do they describe their choice of method—particularly when meta-analysis is possible with included studies? Our meta-analysis was underpowered to consider subgroups and effect modification, but a future analysis including more studies could examine this relationship within and between studies.

## Conclusion

Narrative synthesis and meta-analysis, in this systematic review, yielded similar conclusions, albeit with different strengths and benefits to each method. In conclusion, we would suggest that it is the usage and purpose of the review that should drive the choice of synthesis method. The original report [7], which was designed to inform a specific policy decision, incorporated all relevant evidence in drawing their conclusions. However, other reviews with different uses may find meta-analysis to be the best option in accomplishing their analytic goals.

## Additional file

Additional file 1: Studies not included in the meta-analysis. (DOCX 13 kb)

## Abbreviation

UK: United Kingdom
