# Supplementary material for: Just how plain are plain tobacco packs: re-analysis of a systematic review using multilevel meta-analysis suggests lessons about the comparative benefits of synthesis methods
Source: Syst Rev. 2018 Oct 5;7:153. doi: 10.1186/s13643-018-0821-7 (PMC6173910; doi:10.1186/s13643-018-0821-7)
Supplement: Supplementary file 1 — Studies not included in the meta-analysis. (DOCX 13 kb) [file 13643_2018_821_MOESM1_ESM.docx]

**Online File 1. Studies not included in the meta-analysis**

**No conversion possible**

Bansal-Travers M, Hammond D, Smith P, Cummings KM (2011) The impact of cigarette pack design, descriptors, and warning labels on risk perception in the U.S. Am J Prev Med 40: 674–682. doi:10.1016/j.amepre.2011.01.021

Gallopel-Morvan K, Moodie C, Hammond D, Eker F, Beguinot E, et al. (2012) Consumer perceptions of cigarette pack design: A comparison of regular, limited edition and plain packaging. Tob Control 21: 502–506. doi:10.1136/tobaccocontrol-2011-050079

Hoek J, Gendall P, Louviere J (2009) Tobacco branding and plain packaging: the new frontier in tobacco control? In: American Marketing Association 20^th^ Anniversary Marketing and Public Policy Conference, Madison Hotel, Washington DC, May 28–30.

Moodie C, Ford A, MacKintosh AM, Hastings G (2012) Young people’s perceptions of cigarette packaging and plain packaging: an online survey. Nicotine Tob Res 14: 98–105. doi:10.1093/ntr/ntr136

Rootman I, Flay BR (Principal Investigators), Northrup D, Foster MK, Burton D, et al. (Co-Investigators) (1995) A study on youth smoking: Plain packaging, health warnings, event marketing and price reductions. Toronto, ON: Centre for Health Promotion, University of Toronto. Available: http://www.utoronto.ca/chp/download/RptsandPresents/ youthsmoking.pdf. Accessed 2013 April 8.

**Unextractable outcomes**

Bondy SJ, Paglia A, Kaiserman MJ (1996) Tobacco purchasing and marketing. In: Stephens T, Morin M, editors (Health Canada). Youth Smoking Survey, 1994: Technical Report. Ottawa, ON: Minister of Supply and Services Canada (Catalogue No. H49-98/1-1994E). pp.153–179. Available: http://www.hc-sc.gc.ca/hc-ps/alt_formats/hecs-sesc/pdf/pubs/tobac-tabac/yss-etj-1994/yss_ch-8_1994-eng.pdf. Accessed 2013 April 8.

Centre for Health Promotion (1993) Effects of plain packaging on the image of tobacco products among youth. Toronto, ON: Centre for Health Promotion, University of Toronto. Available: http://legacy.library.ucsf.edu/tid/fuf13d00. Accessed 2013 April 8.

Donovan R (1993) Smokers’ and non-smokers’ reactions to standard packaging of cigarettes. Perth: University of Western Australia. Available: http://legacy.library.ucsf.edu/tid/stl70g00/ pdf. Accessed 2013 April 8.

**Within-subjects designs**

Hammond D, Dockrell M, Arnott D, Lee A, McNeill A (2009) Cigarette pack design and perceptions of risk among UK adults and youth. Eur J Public Health 19: 631–637. doi:10.1093/eurpub/ckp122

Hoek J, Wong C, Gendall P, Louviere J, Cong K (2011) Effects of dissuasive packaging on young adult smokers. Tob Control 20: 183–188. doi:10.1136/tc.2010.037861

Moodie C, MacKintosh AM, Hastings G, Ford A (2011) Young adult smokers’ perceptions of plain packaging: a pilot naturalistic study. Tob Control 20: 367–373. doi:10.1136/tc.2011.042911

Thrasher JF, Rousu M, Hammond D, Navarro A, Corrigan J (2011) Estimating the impact of pictorial health warnings and ‘‘plain’’ cigarette packaging: Evidence from experimental auctions among adult smokers in the United States. Health Policy 102: 41–48. doi:10.1016/j.healthpol.2011.06.003
